# Online teaching in radiology as a pilot model for modernizing medical education: results of an international study in cooperation with the ESR

**DOI:** 10.1186/s13244-021-01092-5

**Published:** 2021-10-19

**Authors:** Fabian Stoehr, Lukas Müller, Adrian P. Brady, Carlo Catalano, Peter Mildenberger, Aline Mähringer-Kunz, Felix Hahn, Christoph Düber, Erkan Celik, Elisabeth Diehl, Pavel Dietz, Daniel Pinto dos Santos, Roman Kloeckner

**Affiliations:** 1 Department of Diagnostic and Interventional Radiology, University Medical Center of the Johannes Gutenberg-University Mainz, Langenbeckst. 1, 55131 Mainz, Germany; 2 Radiology Department, Mercy University Hospital, Grenville Pl, Centre, Cork, T12 WE28 Ireland; 3 Department of Radiology, School of Medicine, University College Cork, Gaol Walk, Cork, T12 YN60 Ireland; 4 Department of Radiological Sciences, Oncology and Anatomical Pathology, Sapienza University of Rome, Piazzale Aldo Moro, 5, 00185 Rome, Italy; 5 Department of Radiology, University Hospital Cologne, Kerpener St. 62, 50937 Cologne, Germany; 6 Institute of Occupational, Social and Environmental Medicine, University Medical Center of the University of Mainz, Langenbeckst. 1, 55131 Mainz, Germany

**Keywords:** Radiology, Medical education, Online education, Curriculum, COVID-19

## Abstract

**Background:**

Due to the outbreak of the coronavirus disease 2019 (COVID-19), it proved necessary to rapidly change medical education from on-site to online teaching. Thus, medical educators were forced to rethink the purpose of teaching and the best form of transmission of knowledge. In cooperation with the European Society of Radiology (ESR), we investigated the attitudes of radiologists in Europe and North America toward innovative online teaching concepts.

**Methods:**

In total, 224 radiologists from 31 different countries participated in our cross-sectional, web-based survey study. On a 7-point Likert scale, participants had to answer 27 questions about the online teaching situation before/during the pandemic, technical and social aspects of online teaching and the future role of online teaching in radiology.

**Results:**

An overwhelming majority stated that radiology is particularly well-suited for online teaching (91%), that online teaching should play a more prominent role after the pandemic (73%) and that lecturers should be familiar with online teaching techniques (89%). Difficulties include a higher workload in preparing online courses (59%), issues with motivating students to follow online courses (56%) and the risk of social isolation (71%). Before the pandemic, only 12% of teaching was provided online; for the future, our participants deemed a proportion of approximately 50% online teaching appropriate.

**Conclusion:**

Our participants are open-minded about online teaching in radiology. As the best way of transferring knowledge in medical education is still unclear, online teaching offers potential for innovation in radiology education. To support online teaching development, a structured, framework-based “online curriculum” should be established.

**Supplementary Information:**

The online version contains supplementary material available at 10.1186/s13244-021-01092-5.

## Key points

- The best way of transferring knowledge in medical education is still unclear.
- Online teaching offers potential for innovation in radiology education.
- The future challenge will be to further develop and integrate novel online teaching concepts.

## Background

The coronavirus disease 2019 (COVID-19) pandemic necessitated radical changes in medical education across the globe. In order to maintain sufficient teaching, innovative online teaching concepts had to be adopted under considerable time pressure [1–4]. However, as a positive consequence of the crisis, medical (and in particular radiological) educators were forced to rethink the “best” way to impart knowledge to medical students [4, 5].

Current studies on this topic have established that both medical students and lecturers are highly interested in innovative concepts including online teaching and learning [6, 7]. Nevertheless, before the pandemic, few medical schools included such innovative concepts in their portfolio [8–10]. In the wake of the pandemic, first attempts to implement different teaching techniques have been made; however, opinions differ on how medical education should be delivered in the future [4, 7, 11, 12].

One positive impact of current pandemic has been that radiologists and medical students were pushed to explore hitherto unknown areas of teaching and learning in medical education. Radiology, as a technologically driven specialty, has an outstanding opportunity to lead in adopting valuable innovative teaching techniques. Consequently, we aimed to evaluate radiologists’ attitudes toward online learning in general, toward “real life” solutions for implementation of online learning, and toward potentially promising teaching concepts after the pandemic. In cooperation with the Education Committee of the European Society of Radiology (ESR), we decided to conduct this study among radiologists in Europe and North America in order to provide a broad scientific basis for future development of teaching and learning in radiology.

## Methods

### Study setup

This survey was conducted as a cross-sectional, web-based study. Primary target group were ESR members who registered themselves as “academic radiologists.” The study has been conducted in accordance with the “Checklist for Reporting Results of Internet E-Surveys (CHERRIES)” [13] and with the “Strengthening the Reporting of Observational Studies in Epidemiology (STROBE) Statement: guidelines for reporting observational studies” [14] (Additional file 1: STROBE checklist). Institutional review board approval was granted by the Ethics Committee of the Medical Association of Rhineland-Palatinate.

### Questionnaire design

A dedicated questionnaire was designed together with the Institute of Occupational, Social and Environmental Medicine (ASU) of the University Medical Center Mainz. The questionnaire consisted of various sections covering in particular the following topics: online teaching situation before and during the pandemic, types of online teaching currently offered and desired in future, technical and social aspects of online teaching, attitudes toward the current and future role of online teaching in radiology, suitability of different concepts for online teaching in radiology, changes in workload due to online teaching and changes in general workload, as well as current and desired ratio between online and on-site teaching. In total, the questionnaire comprised 27 questions. Participants were asked to answer the questions using a 7-point Likert scale (1 = “strongly disagree,” 2 = “disagree,” 3 = “somewhat disagree,” 4 = “neutral,” 5 = “somewhat agree,” 6 = “agree,” to 7 = “strongly agree”). Compared to a 5-point scaling system, a 7-point Likert scale provides a higher variance and thus higher reliability [15]. Nine-point or even 11-point scales would not add any more value regarding the information obtained and could even strain our participants’ abstraction capabilities [16]. Furthermore, the questionnaire contains several questions from the Copenhagen Psychosocial Questionnaire (COPSOQ) as well as from the Prime MD patient Health Questionnaire 4 (PHQ-4). Both questionnaires are validated on a 5-point and a 4-point Likert scale [17, 18]. The entire questionnaire is attached in the Additional file 2: Questionnaire.

### Validation of the questionnaire

The questionnaire underwent a two-step validation approach in order to further enhance the quality of the study. First, cognitive pretesting was performed on a small sample size of 10 participants [19]; second, pilot testing was performed on a larger cohort of 25 participants [20].

### Distribution of the questionnaire

Invitations to take part in the study were distributed by the Education Committee of the ESR via email to all members of the ESR who registered themselves as “academic radiologists.” Additionally, invitations were distributed via social media (e.g., Twitter, LinkedIn, etc.). All invitations contained an identical short introductory text and the hyperlink to access the survey. The participants were informed that the survey results would be anonymous and that they were collected for research purposes only. The survey started on December 7, 2020, and was closed on February 15, 2021.

### Data collection and statistical analysis

Survey results were collected via an established online survey tool (SurveyMonkey®, www.surveymonkey.com). Final survey results were exported from SurveyMonkey® as CSV file and subsequently analyzed using R 4.0.2 (A Language and Environment for Statistical Computing, R Foundation for Statistical Computing, https://www.R-project.org; accessed February 2021). Figures were plotted using the ggplot2 and Likert packages [21]. Mean and standard deviation were calculated to analyze results [22].

## Results

### Participants’ demographics

A total of 224 participants completed the questionnaire. Demographic data recorded were sex (59% male, 41% female), age (mean 48 years), country of current employment, years of professional teaching experience, and academic rank/title (excerpt in Table 1).

**Table 1:** Demographic characteristics of the participants

Sex^a^	Female n (%)	90 (41.4)	N/A n (%) 2 (0.9)
	Male n (%)	130 (58.6)	
Age	Mean y (SD)	47.7 (11.3)	N/A n (%) 9 (0.4)
Professional teaching experience	Mean y (SD)	16.7 (10.5)	N/A n (%) 32 (14.3)
Country of current teaching affiliation	Austria n (%)	5 (2.2)	N/A n (%) 2 (0.9)
	Belgium n (%)	6 (2.7)	
	Bosnia & Herzegovina n (%)	2 (0.9)	
	Bulgaria n (%)	7 (3.1)	
	Croatia n (%)	2 (0.9)	
	Denmark n (%)	5 (2.2)	
	Finland n (%)	5 (2.2)	
	France n (%)	9 (4.0)	
	Germany n (%)	32 (14.3)	
	Greece n (%)	5 (2.2)	
	Hungary n (%)	4 (1.8)	
	Ireland n (%)	7 (3.1)	
	Israel n (%)	4 (1.8)	
	Italy n (%)	12 (5.4)	
	The Netherlands n (%)	10 (4.4)	
	Norway n (%)	4 (1.8)	
	Poland n (%)	3 (1.3)	
	Portugal n (%)	2 (0.9)	
	Romania n (%)	2 (0.9)	
	Russia n (%)	1 (0.5)	
	Serbia n (%)	1 (0.5)	
	Slovakia n (%)	1 (0.5)	
	Slovenia n (%)	1 (0.5)	
	Spain n (%)	19 (8.4)	
	Sweden n (%)	5 (2.2)	
	Switzerland n (%)	9 (4.0)	
	Turkey n (%)	27 (12.0)	
	Ukraine n (%)	1 (0.5)	
	United Kingdom n (%)	14 (6.3)	
	USA n (%)	16 (7.1)	
	Other n (%)	1 (0.5)	
Sex^a^	Female n (%)	90 (41.4%)	N/A n (%) 2 (0.9%)
	Male n (%)	130 (58.6%)	
Age	Mean y (SD)	47.7 (11.3)	N/A n (%) 9 (0.4%)
Professional teaching experience	Mean y (SD)	16.7 (10.5)	N/A n (%) 32 (14.3%)

SD, standard deviation; N/A, not available

^a^Answer options included “non-binary” as well, which was not chosen by any participant

### Survey results

**Fig. 1:**
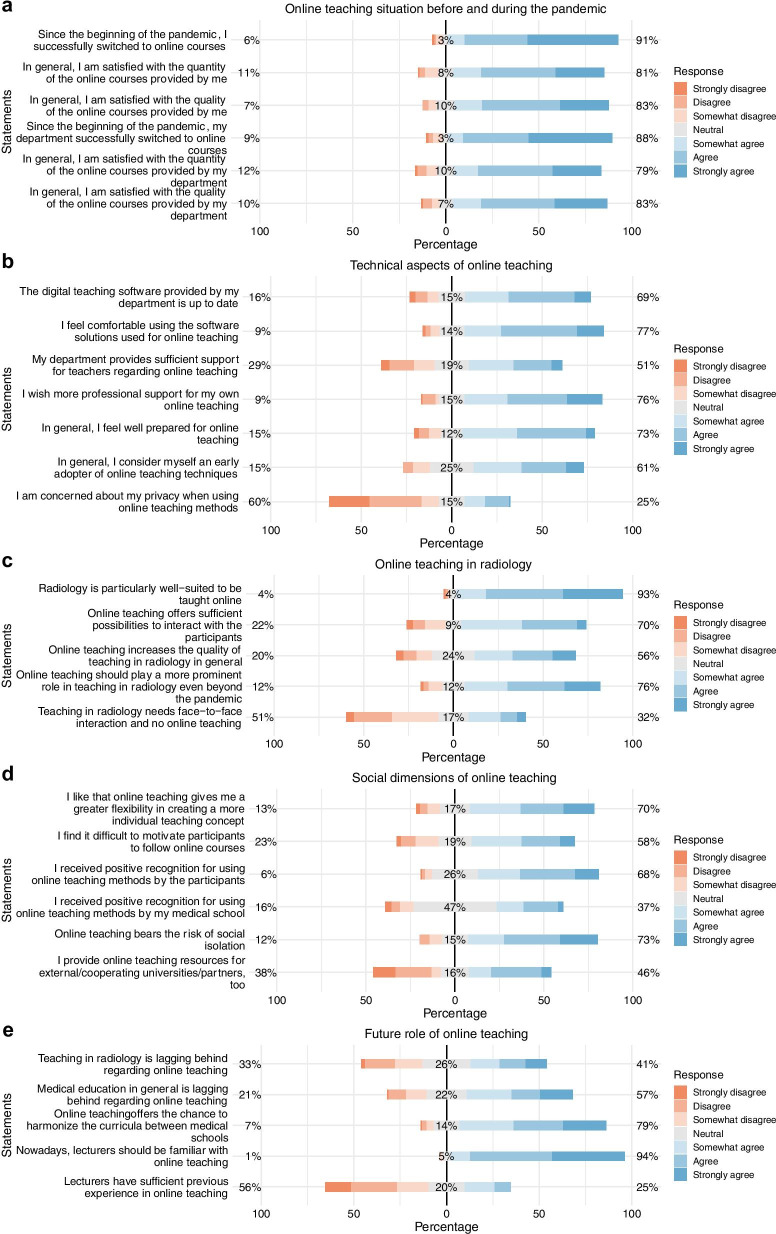
Detailed responses regarding “online teaching situation before and during the pandemic” (**a**), “technical aspects of online teaching” (**b**), “online teaching in radiology” (**c**), “social dimensions of online teaching” (**d**), and “future role of online teaching” (**e**). Orange represents “disagreement,” gray represents “neutral,” whereas blue represents “agreement”

**Fig. 2:**
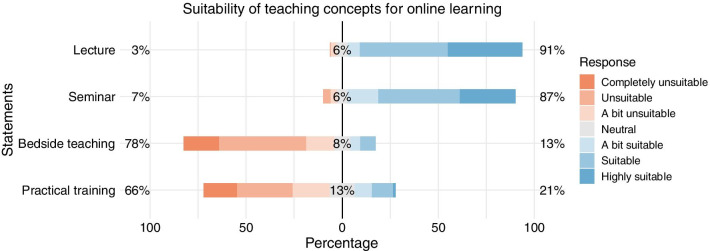
Ranking of teaching concepts being suitable for online learning. It includes four categories and ranges from highly suitable (lecture) to unsuitable (practical training)

**Fig. 3:**
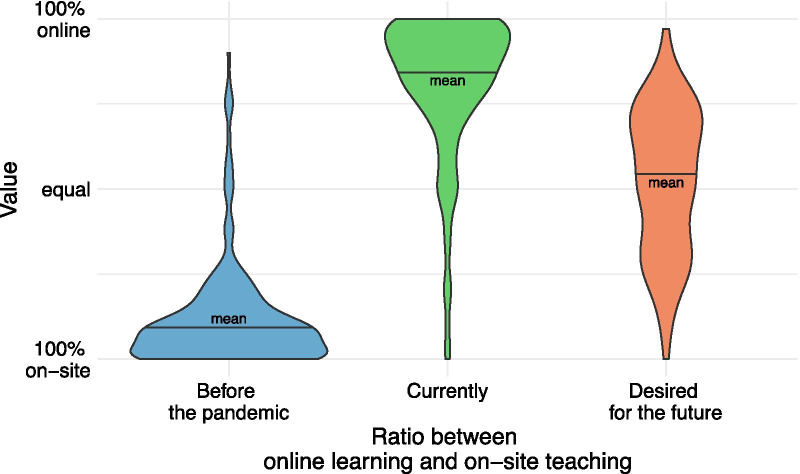
The most left violin plot illustrates the pre-pandemic proportion of online teaching compared to on-site teaching (12%). The middle plot illustrates the current proportion of online teaching compared to on-site teaching (81%) (status during the pandemic). The far-right plot illustrates the proportion of online teaching compared to onsite teaching wished for the future (50%)

In the following, survey results for each particular category are presented in written form as well as graphically (Figs. 1, 2, 3). To increase comprehensibility, the text only contains the key findings and summarizes “strongly disagree,” “disagree” and “somewhat disagree” as disagreement and “somewhat agree,” “agree” and “strongly agree” as agreement. For statistical analyses, the original 7-point categories were used. The entire results can be seen briefly in Fig. 1.

#### Online teaching situation before and during the pandemic (lecturer’s and department’s situation)

Only very few of our participants held online teaching courses before the pandemic (16%). However, most of our participants switched to online teaching during the pandemic (87%). Three out of four were satisfied with both quantity and quality of their online courses (76% and 78%). These results (from a lecturer’s individual perspective) are almost identical with the online teaching situation at the department (Fig. 3).

#### Types of online teaching currently offered and desired in the future

Interactive live lectures were the most common type of teaching offered (88%). Pre-recorded lectures/seminars were also common (58%), followed (after a gap) by online platforms and resources for self-learning and chats (32% and 27%). Only very few participants offered audio podcasts (3%). The ranking regarding the desired online teaching types in the future followed the same order (Fig. 3).

#### Technical aspects of online teaching

Two out of three postulated that the technical infrastructure of their teaching software is up to date (67%). However, only half of our participants were satisfied with the IT-support offered (50%) and three out of four wished for more professional IT-support (73%). Nevertheless, most participants felt comfortable using the online teaching tools offered (75%) and felt well prepared for online teaching in general (70%). Interestingly, around two thirds considered themselves as an early adopter of online teaching techniques (59%) and less than a quarter of our participants were concerned about their privacy when using online teaching methods (24%) (Fig. 3).

#### Online teaching in radiology

Nearly all of our participants stated that radiology is particularly well-suited to be taught online (91%) and most stated that online teaching should play a more prominent role even after the pandemic (73%). Online teaching offers sufficient possibilities to interact with the participants (68%), and it increases the quality of teaching in radiology in general (55%). Only one third of our participants stated that radiological teaching needs face-to-face interaction (31%) (Fig. 3).

#### Social dimensions of online teaching

For two-thirds of the participants, online teaching might provide increased flexibility (67%). About the same proportion received positive recognition for their online courses by medical students (65%), whereas only 41% received positive recognition by their department. For 71% of our participants, online teaching creates a risk of social isolation and about half of our participants had difficulties in motivating their students to follow online courses (56%). Notably, less than a half of our participants stated that they would provide their online teaching resources for external partners (44%) (Fig. 3).

#### Workload due to online teaching

More than half of our participants stated that switching to online teaching led to a higher workload in preparing their courses (59%).

#### General workload and well-being

A great majority stated that they sometimes (45%) or even more often (35%) have an unevenly distributed workload. 45% of our participants stated that they sometimes get behind with their work; 34% stated that they get behind with their work even more often. Around two thirds stated that they sometimes (30%) or more often (36%) have enough time for their work tasks. Two thirds stated that they sometimes (36%) or more often (31%) do not have enough time to complete all work tasks. In summary, a great majority showed no or only mild distress (55% and 28%, respectively), a tenth showed moderate distress (10%), and only a minority showed severe distress (1%).

#### Future role of online teaching

54% of participants believe that medical education in general is lagging behind where it should be regarding online teaching; 52% felt this is the case specifically for radiology teaching. On the other hand, online teaching offers an opportunity to harmonize different curricula (75%). Although lecturers should be familiar with online teaching nowadays (89%), lecturers seem to be in some need of catching up (53%). Switching to online teaching led to unchanged (38%) or even higher participation in courses (36%) (Fig. 3).

#### Suitability of teaching concepts for online teaching

The vast majority of our participants believe that lectures (88%) as well as seminars (83%) seem to be suitable for online teaching. In general, our participants deem practical training as well as bedside teaching rather unsuitable for online teaching (63% and 76%, respectively) (Fig. 2).

#### Pre-pandemic, current and desired ratio between online and on-site teaching

Before the pandemic, our participants estimated a ratio of 12% online teaching compared to 88% on-site teaching. Due to the pandemic, this has now reversed: the current ratio is estimated at around 81% online teaching vs 19% on-site teaching. For the future, the desired ratio seems to settle down at around 50% online and 50% on-site teaching. Regarding the latter question, however, a considerable variability between 25 and 75% desired online teaching can be seen (Fig. 3).

## Discussion

Assembling views of more than 220 academic radiologists in the ESR’s sphere across Europe and North America, for the first time, this study shows a positive attitude toward up-to-date online teaching techniques in radiological education. Participants were convinced that radiology is particularly well-suited to be taught online. Furthermore, participants stated that online teaching in radiology currently lags behind its capabilities, comprising only 12% compared to 88% on-site teaching pre-pandemic. For the future, participants stated that online teaching should play a more prominent role and consider an amount of around 50% of online teaching to be ideal for radiological teaching.

Forced by the outbreak of the pandemic, medical schools had to switch much of their curriculum from on-site to online teaching [1–4]. As only very few medical schools had online teaching arrangements in place before the pandemic, the switch had the potential to cause trouble regarding teaching logistics and infrastructure [8–10, 23]. This study demonstrates that most of our participants were satisfied regarding the overall quality and quantity of online courses provided by themselves and by the department. Nevertheless, it has to be said that at least an equivalent number of participants wished for more professional support for their teaching, e.g., regarding IT-solutions or IT-infrastructure. This perceived deficit may have been caused by the quick and unstructured switch to online teaching and may resolve itself automatically in time. However, this could also be the reflection of a substantial investment backlog regarding up-to-date teaching equipment [6, 23, 24].

Despite all possible teething problems, parts of clinical teaching in radiology—e.g., in the form of case presentations—can quite simply be offered online compared to teaching rounds in internal medicine or the clinical examination in surgery. Furthermore, radiological tools like RIS and PACS are already fully digitized making online case/tumor conferences very easy. This is probably why most of our participants considered radiology to be particularly well-suited for online teaching. However, teaching in radiology is multifaceted and our participants judge digitization in radiology teaching in a differentiated way: They deem lectures and seminars, but not practical training (e.g., endovascular simulator training) suitable for online teaching. Not only our participants but also medical students are highly interested in innovative online teaching techniques [6, 7]. For example, they appreciate the—nowadays—very easy access to broad web-based learning opportunities without the necessity for any physical presence. However, in most medical schools, current teaching is mainly based on traditional, ex-cathedra concepts [8–10]. As a result, in recent years, participation rates of students in formal teaching have been declining [25, 26]. This may indicate students’ discontent with the current teaching system [25, 26]. On the other hand, it might also reflect the dawn of a new teaching era. Our results prove that online teaching is appreciated by medical students, that the participation rates are constant or even higher compared to regular offline courses and that our participants received positive recognition for their online teaching.

Despite these benefits of online teaching, possible weak points should not be forgotten. As stated by two thirds of our participants, one might be an increased risk of social isolation. This includes missing “live” interactions with both lecturers and colleagues [27, 28]. However, the term “social isolation” implies a multidimensional issue, and its effect is undoubtedly further intensified due to mandatory (and several times repeated) self-isolation in most countries. In turn, this leads to a lack of distinction between home and workplace which might further promote a downward spiral of isolation [11].

Following up on this, approximately half of our participants stated that they had difficulties in motivating their students to follow their online courses. Interestingly, recent studies postulate that the teaching type (e.g., problem based vs. lecture based) might affect students’ motivation more than the teaching format (e.g., online vs. on-site teaching) [29, 30]. The difficulty in motivation reported by our respondents can be explained by the fact that the shift toward online learning was performed as “emergency remote teaching” instead of a structured transition. Thus, it can be assumed that most radiology departments did not have a dedicated framework for their online teaching courses. In order to avoid leaving students behind and to sustainably enhance their motivation, future teaching concepts should mainly focus on creating a specific curriculum including schedules and frequent performance reviews.

It is quite understandable that such a venture is not possible without any additional effort. Lectures have to be made “suitable” for online courses, technical infrastructure has to be installed or at least adapted, etc. [23, 24]. Thus, nearly two thirds of our participants stated that preparing online courses was associated with a higher workload. However, it is in the nature of things that establishing a new concept is always associated with additional effort. Furthermore, as the pandemic situation has already lasted more than a year, most former on-site courses have already successfully switched to online. From this *potpourri* of online teaching resources, new opportunities will rise. Flagship projects from recent years showed how digitization of education opens new horizons for teaching and learning. Recently, for example, the German Radiological Society (DRG) launched an interactive web-based learning platform offering a teaching program including online courses, recorded lectures, etc. for medical students (http://conrad.drg.de/). Not only does it allow for easy access to broad knowledge on demand but also it helps to structure knowledge and may even foster the harmonization of educational content in the future.

### Limitations

This study is a questionnaire-based survey and entails typical pitfalls of those. First, there is selection bias, meaning that highly interested or motivated participants are more likely to complete the survey [31]. Next, this study was directed at radiologists, which represent a specialized community within the broader medical community. Even though this group was the particular target of the study, the risk of confirmation bias remains, given that radiologists as technophilic physicians may believe to a greater extent in online teaching than physicians of other subspecialties would do [32]. Furthermore, there is potential social desirability, meaning that participants choose the answer which they assume is favorable [33]. In order to mitigate this kind of bias, we chose a completely anonymous and untraceable study design and instructed the participants that the survey results were for research purposes only. Regarding technical issues, video teleconference tools used by the participants for their online teaching were not standardized and results may be affected using a variety of different tools. Further studies should investigate this issue. Ultimately, we decided on a cross-sectional study design, which allows only for a snapshot. To assess long-term effects, further longitudinal studies are necessary. Such follow-up studies could focus on which concepts or combinations are most suitable to impart knowledge to medical students. This could provide a more in-depth analysis of different teaching types. Further studies could also investigate differences in how lecturers and medical students see “their” future of medical education. Further studies could also investigate if online teaching might foster the harmonization of teaching curricula around the world. As this study mainly focused on industrialized countries across Europe and North America, further studies could include emerging or developing countries in order to more deeply analyze socioeconomic factors in teaching radiology.

## Conclusion

Summarizing the results of our study, the overarching challenge should be to design a dedicated, new curricular framework by reasonably integrating innovative online teaching concepts. The pandemic has forced the whole medical community to rethink the processes of learning and the best ways to impart knowledge to medical students. Thus, we are convinced, after the pandemic is over medical education will have changed substantially. Furthermore, we strongly believe that the lessons we have learned from 2020 will further stimulate beneficial changes in teaching. As developing, implementing, and improving innovative techniques is at the heart of radiology, we are convinced that the current situation creates an extraordinary opportunity for radiology to become a pioneer in modernizing medical education.

## Supplementary Information

**Additional file 1.** Guidelines for reporting observational studies.

**Additional file 2.** Questionnaireinvestigating various aspects of online teaching in medical education and radiology.

## Data Availability

The datasets used and/or analyzed during the current study are available from the corresponding author on reasonable request.

## Abbreviations

COVID-19: Coronavirus disease 2019
ESR: European Society of Radiology
STROBE: Strengthening the Reporting of Observational Studies in Epidemiology
CHERRIES: Checklist for Reporting Results of Internet E-Surveys
ASU: Institute of Occupational, Social and Environmental Medicine
