# Supplementary material for: Online teaching in radiology as a pilot model for modernizing medical education: results of an international study in cooperation with the ESR
Source: Insights Imaging. 2021 Oct 19;12:141. doi: 10.1186/s13244-021-01092-5 (PMC8524216; doi:10.1186/s13244-021-01092-5)
Supplement: Supplementary file 2 — Additional file 2. Questionnaireinvestigating various aspects of online teaching in medical education and radiology. [file 13244_2021_1092_MOESM2_ESM.docx]

**ELECTRONIC SUPPLEMENTARY MATERIAL**

Dear colleagues!

We are highly interested in your knowledge about online teaching in radiology and we appreciate your feedback! The survey should take only 5 minutes to complete.

Of course, your participation is voluntary, and your answers will be anonymous and untraceable. Answers will be collected for research purposes only.

Thank you very much for your help!

The study team on behalf of the Educational Committee of the ESR

# Gender


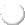
 Male
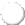
 Female


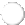
 Non-binary

# Age

1. **Country where I currently teach in**


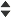


# Professional teaching experience in years (including all teaching types, e.g. lectures, seminars, etc.)

1. **Academic rank/title**


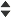


# Most often, I teach...


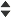


1. **Online teaching before the COVID-19 pandemic from my perspective:**

Yes No


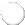

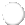


**Before** the **beginning** of the pandemic, **I already oﬀered online courses**.

# Online teaching during the COVID-19 pandemic from my perspective:

Strongly

disagree Disagree

Somewhat

disagree Neutral

Somewhat

agree Agree

Strongly agree

I do not know/not applicable


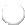

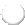

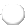

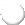

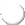

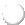

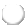

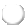


**Since** the **beginning** of the pandemic, **I** successfully **switched to online courses**.


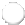

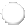

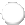

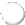

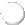

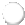

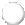

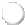
In general, I am satisﬁed with the **quantity** of the **online courses** provided **by me.**


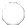

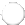

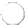

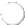

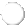

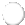

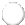

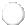


In general, I am satisﬁed with the **quality** of the **online courses** provided **by me**.

# The following types of online teaching are currently oﬀered by me (multiple answers possible):


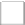
 Interactive live lectures or seminars ("webinars")


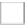
 pre-recorded lectures or seminars without possibility of interaction


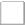
 Online platforms and resources for self-learning/self-assessment modules
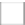
 Chats (with/without video stream)


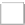
 Audio podcasts


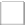
 I do not know/ not applicable
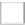
 Other:

# In future, I would like to oﬀer the following types of online teaching methods. Please rank via "drop down" from 1-5 (1=most preferred, 5=least preferred):


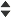

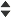

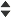

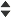

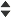


o

Interactive live lectures or seminars (webinars)

o

Pre-recorded lectures or seminars without possibility of interaction

o

Online platforms and resources for self-learning/self-assessment modules.

o

Chats (with/without video stream)

o

Audio podcasts

1. **Online teaching before the COVID-19 pandemic at my department:**

Yes No


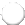

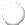


**Before** the **beginning** of the pandemic, **my department already** oﬀered **online courses**.

# Online teaching during the COVID-19 pandemic at my department:

Strongly

disagree Disagree

Somewhat

disagree Neutral

Somewhat

agree Agree

Strongly agree

I do not know/not applicable


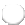

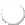

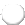

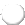

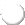

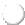

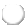

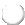


**Since** the **beginning** of the pandemic, **my department** successfully **switched** to **online courses**.

In general, I am satisﬁed with the **quantity** of

the **online
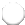
**
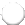

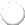

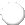

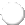

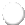

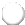

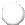
 **courses**

provided **by my department**.


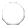

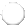

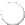

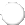

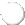

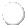

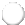

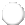


In general, I am satisﬁed with the **quality** of the **online courses** provided **by my department**.

# The following types of online teaching are currently oﬀered by my department (multiple answers possible):


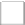
 Interactive live lectures or seminars ("webinars")


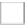
 pre-recorded lectures or seminars without possibility of interaction


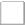
 Online platforms and resources for self-learning/self-assessment modules
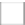
 Chats (with/without video stream)


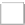
 Audio podcasts


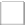
 I do not know/not applicable
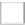
 Other:

# Technical aspects of online teaching at my department:

Strongly

disagree Disagree

Somewhat

disagree Neutral

Somewhat

agree Agree

Strongly agree


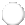

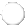

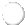

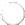

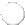

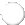

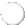


The **digital teaching software** provided by my department is **up to date**.


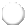

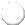

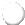

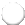

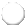

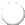

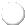
I **feel comfortable using the software** solutions used for online teaching (e.g. Microsoft Teams, Moodle, Zoom, Skype, etc.).


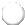

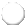

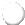

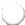

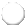

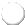

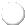


My **department** provides **suﬃcient support** for teachers regarding **online teaching** (e.g. by IT department).

## I wish more professional

**support** for my
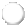

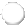
 own online

teaching.

In general, I feel **well prepared** for online teaching.

# All in all, I rank the quality of my department's technical infrastructure for online teaching:

Extremely bad

50%/50%

Extremely good

1. **Online teaching in radiology:**

Strongly

disagree Disagree

Somewhat

disagree Neutral

Somewhat

agree Agree

Strongly agree

**Radiology** is particularly **well- suited** to be taught online (e.g. case-based conferences, etc.).

## Online teaching oﬀers suﬃcient

possibilities to **interact** with the

## participants.

**Online teaching increases** the **quality of teaching in radiology** in general.

**Online teaching** should play a **more prominent**

**role in teaching**  **in radiology** even

beyond the pandemic.

**Online teaching** bears

the **risk** of **social isolation**.

Teaching in **radiology needs face-to-face interaction** and no online teaching.

# My involvement with online teaching:

Strongly

disagree Disagree

Somewhat

disagree Neutral

Somewhat

agree Agree

Strongly agree

**I like** that online teaching gives me a **greater ﬂexibility** in creating a more individual teaching concept.

I ﬁnd it **diﬃcult** to **motivate**

**participants** to follow online courses.

I received **positive recognition** for using online teaching methods by the **participants**.

I received **positive recognition** for using

online teaching methods by my

## medical school.

I provide **online teaching resources** for

external/cooperating **universities**/**partners**, too (e.g., recorded lectures, seminars, etc.).

I am concerned about **my privacy** when using online teaching methods.

In general, I consider myself an **early adopter** of

online **teaching techniques**.

# My workload due to online teaching:

Much less Less A bit less Neutral A bit higher Higher Much higher

**Regarding yourself**, did switching to online teaching lead to a **lower or higher amount of workload** to prepare your courses?

# Regarding your "general" workload (regardless of online teaching):

Always Often Sometimes Seldom Never/hardly ever

Is your **workload unevenly** distributed so it piles up?

Do you **get**

**behind** with your work?

Do you have **enough time** for your work tasks?

How often do you **not have**

**time** to complete all your work

tasks?

# Regarding your general well being: Over the last 2 weeks, how often have you been bothered by the following problems?

Not at all Several days

More than half the

days Nearly every day

Feeling **nervous**, **anxious** or **on edge**?

Not being able to

stop or control **worrying**?

**Little interest** or **pleasure** in doing things?

Feeling **down**,

**depressed**, or **hopeless**?

# The future of online teaching:

Strongly

disagree Disagree

Somewhat

disagree Neutral

Somewhat

agree Agree

Strongly agree

**Teaching in radiology** is **lagging**

**behind** regarding online teaching.

**Medical education** in general is **lagging**

**behind** regarding online teaching.

**Online teaching** oﬀers the chance to **harmonize** the **curricula** between **medical schools**.

Nowadays, **lecturers should be familiar** with online teaching.

**Lecturers** have **suﬃcient previous experience** in online teaching.

# Switching to online teaching:

Much less Less A bit less Neutral A bit higher Higher Much higher

**Regarding your students**, did switching to online teaching lead to a **lower or**

**higher participation** in courses?

# How much are the following teaching concepts suitable for online teaching?

Completely

unsuitable Unsuitable

A bit

unsuitable Neutral

A bit

suitable Suitable

Highly suitable

Lecture

Seminar

Bedside teaching

Practical training,

e.g. simulator training

# Before the pandemic, the mix between on-site and online teaching was:

On-site (100%)

Equal mix (50%/50%)

Online (100%)

1. **The current mix between on-site and online teaching is:**

On-site (100%)

Equal mix (50%/50%)

Online (100%)

# In future, the perfect mix between on-site and online teaching is:

On-site (100%)

Equal mix (50%/50%)

Online (100%)
